# A focus group study to understand biases and confounders in a cluster randomized controlled trial on low back pain in primary care in Norway

**DOI:** 10.1186/s12875-018-0759-9

**Published:** 2018-05-22

**Authors:** Erik L. Werner, Ida Løchting, Kjersti Storheim, Margreth Grotle

**Affiliations:** 1 Department of General Practice, Institute of Health and Society, Faculty of Medicine, University of Oslo, Oslo, Norway; 2 Research and Communication Unit for Musculoskeletal Health (FORMI) Clinic for Surgery and Neurology, Oslo University Hospital, Oslo, Norway; 3 Department of Physiotherapy, Oslo Metropolitan University/FORMI, Clinic for Surgery and Neurology, Oslo University Hospital, Oslo, Norway

## Abstract

**Background:**

Cluster randomized controlled trials are often used in research in primary care but creates challenges regarding biases and confounders. We recently presented a study on low back pain from primary care in Norway with equal effects in the intervention and the control group. In order to understand the specific mechanisms that may produce biases in a cluster randomized trial we conducted a focus group study among the participating health care providers. The aim of this study was to understand how the participating providers themselves influenced on the study and thereby possibly on the results of the cluster randomized controlled trial.

**Methods:**

The providers were invited to share their experiences from their participation in the COPE study, from recruitment of patients to accomplishment of either the intervention or control consultations. Six clinicians from the intervention group and four from the control group took part in the focus group interviews. The group discussions focused on feasibility of the study in primary care and particularly on identifying potential biases and confounders in the study. The audio-recorded interviews were transcribed verbatim and analyzed according to a systematic text condensation. The themes for the analysis emerged from the group discussions.

**Results:**

A personal interest for back pain, logistic factors at the clinics and an assessment of the patients’ capacity to accomplish the study prior to their recruitment was reported. The providers were allowed to provide additional therapy to the intervention and it turned out that some of these could be regarded as opposed to the messages of the intervention. The providers seemed to select different items from the educational package according to personal beliefs and their perception of the patients’ acceptance.

**Conclusion:**

The study disclosed several potential biases to the COPE study which may have impacted on the study results. Awareness of these is highly important when planning and conducting a cluster randomized controlled trial. Procedures in the recruitment of both providers and patients seem to be key factors and the providers should be aware of their role in a scientific study in order to standardize the provision of the intervention.

**Electronic supplementary material:**

The online version of this article (10.1186/s12875-018-0759-9) contains supplementary material, which is available to authorized users.

## Background

The nature of primary health care creates challenges for clinical trials that are different from the specialized health care. A limited number of eligible patients at each site in primary care require a greater number of clinicians at different locations involved in the trial than in a hospital department with referred patients.

Therefore cluster randomized controlled trials (cRCT) are often used in primary care settings because the randomization procedure is then performed on the health care providers and not on the subjects producing the data [1]. However, both ethics and validity of the data have been questioned in the cRCT study design [2, 3].

We have recently conducted and published a study on a treatment of low back pain (LBP) among general practitioners (GPs) and physiotherapists (PTs) in Norway, using cRCT as the study method [4]. The COPE study aimed to produce a simple tool for GPs and PTs in primary care for treatment of patients with LBP. The providers in the intervention group were thoroughly trained for performing the intervention appropriately, while the providers in the control group were instructed to follow their best practices as usual care. Also in the intervention group, additional care according to the provider’s or the patient’s wish was allowed (i.e. physiotherapy, medication etc.) [5].

Although we observed great improvements in both disability and sickness absence in both the intervention group and the control group, we were unable to report significant differences between the groups [4]. We therefore wanted to investigate whether there were factors related to the study design that were accountable for this result. In scientific literature such process evaluation are more often asked for [6].

As opposed to a randomized controlled trial (RCT) where the subjects in question are facing a randomization procedure, the cRCT involves an extra level [1]. The randomization procedure is normally conducted on a number of providers who in turn will provide the intervention on the subjects who produce the data. While an intervention in studies on drugs may be easily accomplished regarding blinding and administration and dosage of the actual drug in study, this is more complicated when people constitutes the intervention. This may be even more complicated when the health care providers involved are not working at the same department and have different backgrounds, experiences and attitudes. How may we ensure that the subjects actually receive an identical intervention provided by different providers at different locations?

The challenges with differences between providers providing a specific procedure in clinical practice may also be valid in ordinary RCTs conducted at different locations or by different providers as in multicenter studies. However, we assumed that the extra level of the recruitment procedure (providers and patients) in a cRCT poses more possible biases to the study. Therefore, we invited all participating clinicians in the COPE study [4] to a focus group discussion on this theme. The aim of this study was to understand how the participating health care providers themselves influenced on the study and thereby possibly on the results.

## Methods

### The COPE study

In 2008 20 general practitioners (GPs) and 20 physiotherapists (PTs) were recruited through an information meeting at Oslo University Hospital Ullevål, to participate in a clinical trial in primary health care on LBP patients. Through a randomization procedure, half of the GPs and PTs were randomized to perform the intervention while the other half constituted the control group. All providers should include consecutively LBP patients with more than 4 weeks duration of pain, but less than 1 year. All patients in both intervention and control group would meet with their provider for 30 min every week for 4 weeks, but only those in the intervention group would be provided with the specific content of the trial.

A manual based on the “Explain Pain” concept of Butler & Moseley [7] was developed adjusted to the setting and aims of the study. The “Explain Pain” concept is a simple education of patients in pain physiology, and the idea is that if the LBP patient understands these mechanisms he or she will be better capable to draw right conclusions on health behavior and consequently cope with the pain until it eventually fades away [7]. The providers in the intervention group were thoroughly trained in the manual while the concept was not communicated to the control group. The protocol for the study is previously described in detail [5] and the results are presented elsewhere [4].

According to the protocol, the recruitment of 300 patients was planned to take place during 2009 and 2010. For several reasons, the recruitment period lasted for 4 years and was not closed until January 2013 with 220 included patients [4].

### The focus group study

We conducted a generic qualitative study as the aim of our study was to understand the processes of the COPE study [8, 9]. All 36 participating clinicians in the COPE study were in spring 2013 invited by e-mail or a phone call to take part in a focus group study on their experiences with the study. Six clinicians from the intervention group and four from the control group participated in two separate focus group interviews. Four of the participants from the intervention groups were PTs while two were GPs. In the control group, three of the participants were PTs while one was a GP. Each of the two group sessions lasted for 90 min (the participants were gathered only once) and took place at the location of the Research and communication unit for musculoskeletal health (FORMI) at Oslo University Hospital.

The participants were invited to share their experiences from their participation in the COPE study, from recruitment of patients to accomplishment of either the intervention or control consultations. In the group discussions we focused on feasibility of the study in primary care and particularly on identifying potential biases and confounders in the study. An interview guide was produced prior to the interviews and the groups were moderated by ELW with MG and IL as observers (see Additional file 1). ELW is experienced by three former qualitative studies (of which two focus group studies) while MG and IL had limited experience with this methodology. The themes of the interview guide followed each step of the study from recruitment and randomization of health care providers, to recruitment of patients at each site. We also discussed the content of the four sessions in both the intervention and the control groups. Also the providers’ adherence to the manual in the intervention group was discussed and for all participants, their personal views on the study were asked for.

The audio-recorded interviews were transcribed verbatim and all authors collaborated on analysis conducted as systematic text condensation [10]. The four steps comprised: (i) reading all the material to obtain an overall impression, (ii) identifying units of meaning, representing different aspects of participants’ experiences with the study according to the aims of the study, and coding for these, (iii) condensing the contents of each of the coded groups, and (iv) summarizing the contents of each group to generalize the findings.

The systematic text condensation starts with all researchers reading all the audio-recorded interviews separately. Following a discussion of these, we started to group statements and experiences into units of meaning. Then we looked at our units and found several that could be combined. The coding procedure had the four steps of the COPE study as starting point and units of meaning were placed according to these steps. We thoroughly checked that all important statements found their place in one of the categories.

### Ethics

All GPs and PTs participating in the COPE study were invited to take part in the focus group study. We included all those who volunteered and due to the limited number of participants we were unable to balance the groups according to age, gender, years of practice. The participants were informed about the study and gave their consent orally. We found the study not to be subject to approval by the Ethical Committee due to the participants’ professional status. The tape recordings and the transcriptions are all anonymized and stored at FORMI, Oslo University Hospital.

## Results

The results were categorized into four important aspects of the accomplishment of the COPE study: (i) recruitment of participating clinicians, (ii) recruitment of participating patients, (iii) conducting the intervention (or control), and (iv) the health care providers’ own benefits from having participated in the study. Within all these aspects, the responses were analyzed in respect of any impact on the study results.

### Specific interest in back pain among the participating professionals

All participants agreed that a genuine interest for back pain was an absolute requirement for their participation. To be a part of research and being in an environment that shared the same interest was described as exciting and interesting. Several also found it developing and felt that participation increased their knowledge about back pain. Several also highlighted that the intervention gave them a practical tool for their management of LBP patients.

*“It was exciting with a new way to address back pain. That was my motivation.” (GP in intervention group).*

### Selections based on good chemistry?

Recruitment of patients was reported to be facilitated by the fact that participation provided four sessions with the provider free of charge. In addition, it was necessary that the provider felt a good chemistry with the patients and that the provider himself or herself had confidence in the content of the intervention.

Due to slow recruitment process of the patients, a small number of patients were recruited through advertising in newspapers during the study period. This was also appreciated by several of the participants, and it seemed that patients recruited through this advertising were more prepared for an intervention containing education rather than physical treatment.

*“It was fantastic to have these that were recruited actively, versus those we picked ourselves. Some of these, they had….they took all the points, they were so ready for it.” (GP in intervention group)*

Some providers disclosed that lack of good chemistry with a patient could exclude patients from the study because the clinician was uncomfortable with the idea of spending so much time with the patient, or was unsure of the patient’s acceptance of the messages. In addition, the providers seemed to conduct an active selection of patients according to their own perceptions of the patient’s language skills and intellectual capacity. It was assumed that the intervention required a certain level of understanding of the messages.

Also general logistics at the clinic seemed to influence the recruitment. Sometimes the secretary or colleagues forgot to refer eligible patients, but also if it was late on the working day the provider felt it too bothersome to inform and in some way persuade the patient to take part in the study.

For some reason all participating providers experienced a general lack of eligible patients which made recruitment slow and difficult. They also experienced the intervention as more demanding and time consuming than ordinary treatment, which may have declined the interest for recruiting patients.

### Difficult to adhere to the manual accurately

Most of the clinicians experienced that providing the intervention was easier across time, indicating improved skills with exercise. They were also encouraged if they experienced that the patient really benefited from the education and improved in health. Some started to use elements of the treatment also for patients with other unspecific pain conditions. However, despite several meetings and a DVD providing accurate accomplishment of each of the four sessions, some of the participants called for more training in the intervention.

According to the participants, patients’ expectancies to the treatment could be both a facilitator and an obstacle. If the patient expected a physical treatment, they could sometimes have a hard time to accept a treatment based on education. Several professionals presented this as specifically challenging. On the other hand, some patients seemed to receive a new understanding of their pain and were grateful for the new coping strategy, which in some way could give more energy to the sessions.

*“The patients expected a treatment – a new treatment. And so they came and received conversations…..about matters they may be already knew. They expected more. And all we said, they did it already….” (GP in intervention group)*

Some of the PTs provided manual therapy as an additional care to the intervention, and the clinicians were asked if this could represent a conflicting communication. On the one hand to explain the pain within the frames of expectations and sensitization and on the other hand provide manipulation on a perceived physical cause for the pain. The clinicians argued with referral to the patients’ expectations and wishes and regarded the manipulation as more complementary treatment than conflicting messages.

One GP illustrated the difficulties with the provision of the messages in the space between psychological and physical aetiology:

*“I think that across time one moved within a very narrow space between somatic and psychological and cognitive….. As “no, it is nothing mental”, “no, you’re not depressed”, “no, there is no injury in the back”. You should, in a way, wedge the message between all these things.” (GP in intervention group)*

Some providers were unsure of their personal qualifications and authority to provide the intervention properly. The discussions even revealed some uncertainty among the providers themselves whether they believed in the messages. Across time, it seemed that many providers selected some of the messages to be included and others, that were perceived too difficult, were excluded.

*“The messages (of the study) seem to be in an outer edge, it is very popular to believe that the cause of the pain is not local but sensitization of the central nerve system…. But there might be cases of combinations….. patients may have a local problem with their recruitment of muscles that may provoke a local pain….” (PT in intervention group).*

While some PTs seemed to experience the 30 min intervention to be insufficient for conducting the study because many patients also wanted additional physical treatment, many of the GPs felt the intervention were too much time consuming. Particularly in the control group the GPs had difficulties with talking about the back pain for 30 min every week.

*“…so when they met at the meetings, you start with the back and “how are you doing” etc. etc.. But I had to be aware not to put any cognitive approach into it because this should be a control group. (…..) So we did a little chatting, yes.” (GP in control group)*

#### Making a strategy for the management of a complex group of patients

All intervention providers participating in the focus group interviews underscored the value of transferring elements of the intervention to other patients with unspecific pain conditions. They also appreciated the new knowledge they had received, which gave them tools for management of a variety of patients. They felt they had increased their consciousness on wordings in general in their communication with patients, and the long term contact with the patients had particularly for the GPs provided a broader insight in the patient’s life.

*“I have learned a lot about pain. When you explain, you learn for yourself…you have to think about what you say. (….) …. Which I probably use when I talk with patients.” (PT in intervention group)*

*“Previously, pain was something that I did not have any tool for talking about or address or explain, but now… I believe I can defuse the pain to a great extent.” (PT in intervention group).*

On the other hand, the providers of the control groups hardly found any benefit from their participation a part from the award of having free training in the intervention after the closure of the study.

## Discussion

This focus group study has disclosed a number of challenging factors that may impact on the results of a cRCT in primary care. The most important one is probably the great possibility of a selection bias. The findings might indicate that some of the clinicians did not include patients from the trial if they did not believe that he or she would be able to accept the content of the messages. Also simple logistic factors at the clinics could result in a selection bias in recruitment of the patients.

The risk of selection bias in cRCT is well known from previous research [11]. Farrin et al. argue that individuals should be recruited to the study before randomizing the professionals as this procedure will more likely ensure equality between the groups [12]. If that is not possible, the cluster allocation could be performed by someone independent by the study [13]. Neither of these procedures could have been possible in the COPE study as the recruitment was based on the providers’ patient population and the patients were recruited consecutively as they visited their provider for back pain. This procedure was chosen in order to have the trial setting as close to normal practice as possible, as development of a tool for the management of back pain in primary care was the aim of the COPE study [4]. However, information according to a risk of bias tool in reporting from a cRCT is helpful to the reader to assess the validity of the study [14].

The participating health care providers also highlighted the importance of their personal interest for LBP and the felt need of a tool for best management of this patient group as necessary for their own enthusiasm and interest for learning this treatment strategy. This possible selection bias both on recruitment of patients and also on the recruitment of health care providers to the study questions the external validity of the study.

On the one hand, the possible selection bias on the recruitment of health care provider to the study may reflect real life: best practice is most likely provided by those with interest and knowledge in the field. However, this may complicate the delivery of the messages of a trial as these may be colored by the providers’ personal attitudes and knowledge to a greater extent than by providers not particularly familiar with the field. This is somewhat exemplified by the PTs offering manual therapy as additional therapy, which was accepted in the study protocol, but still is based on a different view on back pain, being a physical injury in need for specific therapy. Furthermore, several of the PTs in the control group were familiar with the concept of Explain Pain and may unwillingly have provided messages in line with this.

Also adherence and compliance with the trial manual in the COPE study was questioned in the present study. Strategies for improving compliance among the providers have been presented [15]. Such strategies comprise thorough education of the participants not only on the intervention but also about their role as research participants, having continuous contact with the providers, and the provision of incentives for participation. Regarding the COPE study the providers’ understanding of their role as research participants may have been insufficient according to the findings of the present study. The incentives provided (refund of the actual patient cost) may have been too small.

Process evaluation is highly recommended in the literature on cRCTs [15]. Obviously it would be helpful in the management of the study. For the COPE study the continuing meetings with the providers and the provision of a DVD recording the actual delivery of the messages may be regarded as a process evaluation. Following the trial, two separate master thesis have demonstrated a difference in ability to recall the messages of the sessions among the patients, in favor of those in the intervention group, and that the patients found the messages in the two groups equally favorable.

How much training is needed for the providers to be able to conduct a trial in accordance with the study manual? There is no accurate answer to that. In a study comparing three different training conditions, statistically significant differences favoring a seminar plus supervision over review of a manual or manual plus a training web site, were found [16]. The providers in the COPE study underwent an initial seminar and several meetings throughout the study period in addition to a DVD presenting the sessions. They did not have a specific individual supervision [4, 5].

Difficulties with patient recruitment in primary care studies have been reported previously. Therefore a sub-type of cRCT named professional-cluster trial has been suggested [17, 18]. Also in this design, the providers are the target of the intervention but they do not provide any specific intervention towards their patients. It is the impact of the intervention on the providers that may alter their treatment and management of their patients across time that possibly will produce a better outcome on the patients.

Applied on our study, a professional-cluster trial could be that a number of health care providers received a thorough training in the guidelines for the management of LBP. They would not have to recruit patients specifically to a trial but outcomes on patients with LBP could be measured retrospectively at a later stage. The advantage of this design is that the providers do not have to actively recruit patients and by this the selection bias in recruitment is avoided. Possibly it would also have been more in accordance with the ethics suggested by the Ottawa Statement on the Ethical Design and Conduct of Cluster Randomized Trials [19]. A recent study on improved antibiotic prescription among Norwegian GPs has demonstrated success with this method [20]. In this study, the GPs were trained in groups to reduce inappropriate prescribing for older patients. Data was collected through extraction from the GPs electronic patient record system and no patients were actively recruited to the study [21].

There are limitations with this study. One is a lack of data saturation due to a limited number of participants in the focus groups. Although we invited all providers who had participated in the COPE study, only a few were willing to spend time on this. Also only three GPs were attending the groups. Nevertheless we believe that most items were disclosed during the group talks. Furthermore, aiming to explore possible biases in the COPE study by a qualitative study, the systematic text condensation does not necessarily demand “a complete description of all aspects of the phenomenon we study” [10].

For the analysis of this focus group study we chose to follow Malterud’s systematic text condensation [10]. The procedure is presented in the Method section. The aim of the study was to explore the participants’ views on the conduct of the COPE study, in order to understand the results of that trial. A methodology based on phenomenology seemed to be appropriate to that purpose.

## Conclusion

Although the research design cRCT seems somewhat ideal for the structure of primary health care, our experiences disclose several potential serious biases which may be valid for all RCTs but possibly even more for cRCTs. Particularly it seems to be selection biases and uncertainty about the messages delivered. In our opinion the ways to overcome these biases suggested at present in the literature do not easily comply with the infrastructure of primary care. Researchers planning cRCT should pay attention to tools like the Cochrane risk of bias tool, the PRECIS-2 tool and The Ottawa statement on the ethics of cRCTs.

## Additional file

Additional file 1: The Interview Guide provides information on the different topics that were brought into the discussions by the interviewers, which may not be identical to the topics that resulted from the focus groups. (DOCX 15 kb)

## Abbreviations

COPE: The COgnitive patient education trial
cRCT: Cluster randomized controlled trial
FORMI: Research and communication unit for musculoskeletal health
GP: General practitioner
LBP: Low back pain
PT: Physiotherapist
RCT: Randomized controlled trial
