# Supplementary material for: A focus group study to understand biases and confounders in a cluster randomized controlled trial on low back pain in primary care in Norway
Source: BMC Fam Pract. 2018 May 22;19:71. doi: 10.1186/s12875-018-0759-9 (PMC5964728; doi:10.1186/s12875-018-0759-9)
Supplement: Supplementary file 1 — The Interview Guide provides information on the different topics that were brought into the discussions by the interviewers, which may not be identical to the topics that resulted from the focus groups. (DOCX 15 kb) [file 12875_2018_759_MOESM1_ESM.docx]

**INTERVIEW FOCUS GROUP GUIDE – INTERVENTION GROUP**

The purpose of this group discussion is to reveal information to understand the results of the COPE trial.

- About the participants
- Why did you choose to take part in this study?
- Recruitment
- How did you recruit patients? Any specific technics?
- Who did you not ask to take part in the study?
- Conduct of the treatment (the intervention)
- Was it easy or difficult to learn the content of the treatment?
- Were the messages credible and easy to communicate to the patients?
- How did you manage doubt or no response from the patients?
- Were different parts of the intervention addressed differently?
- Did your provision of the messages change across time?
- To what extent did you adjust the messages according to the patient’s response (interest, accept)?
- Characteristics at patients who more easily / difficult accepted the messages
- Characteristics at providers necessary for “selling” the messages?
- Personal perception of the content of the intervention
- Which are your personal perceptions to the content of the “Explain Pain” model / COPE intervention? Do you believe it is correct to treat all unspecific, longlasting back pain patients with this cognitive model?
- Which was the most important message in the COPE intervention (regarding all four sessions)?
- The personal usefulness of participation / educational gain
- Did you learn anything g from your participation in the trial – if so, what?
- Is this a content, or a way to address patients, that you can use at other patients?
- General comments to the COPE trial

**INTERVIEW FOCUS GROUP GUIDE – CONTROL GROUP**

The purpose of this group discussion is to reveal information to understand the results of the COPE trial.

- About the participants
- Why did you choose to take part in this study?
- Recruitment
- How did you recruit patients? Any specific technics?
- Who did you not ask to take part in the study?
- Conduct of the treatment (the control)
- What was the content of your treatment of the COPE patients?
- Did the patient address any objections towards the concept of several appointments without any specific content?
- Did the patients express satisfaction with having the opportunity to see you several times?
- Did the treatment change across time?
- To what extent did you adjust the messages according to the patient’s response (interest, accept)?
- Did you have trouble with conducting appointments without any specific content?
- The personal usefulness of participation / educational gain
- Did you learn anything g from your participation in the trial – if so, what?
- Is this a content, or a way to address patients, that you can use at other patients?
- General comments to the COPE trial
